# Impact of a passive upper-body exoskeleton on muscle activity, heart rate and discomfort during a carrying task

**DOI:** 10.1371/journal.pone.0287588

**Published:** 2023-06-23

**Authors:** Gabriela Garcia, Paul Gonzalo Arauz, Isabel Alvarez, Nicolas Encalada, Shirley Vega, Bernard J. Martin

**Affiliations:** 1 Departamento de Ingeniería Industrial, Colegio de Ciencias e Ingenierías, Universidad San Francisco de Quito USFQ, Quito, Ecuador; 2 Departamento de Ingeniería Mecánica, Colegio de Ciencias e Ingenierías, Universidad San Francisco de Quito USFQ, Quito, Ecuador; 3 Department of Industrial and Operations Engineering, University of Michigan, Ann Arbor, Michigan, United States of America; Kennedy Krieger Institute/Johns Hopkins University School of Medicine, UNITED STATES

## Abstract

**Objective:**

The goal of this study was to compare erector spinae muscle fatigue, upper limb muscle activity, body areas discomfort, and heart rate during a 10-min carrying task with and without a passive upper-body exoskeleton (CarrySuit^Ⓡ^) while considering sex influences.

**Background:**

Passive exoskeletons are commercially available to assist lifting or carrying task. However, evidence of their impact on muscle activity, fatigue, heart rate and discomfort are scarce and/or do not concur during carrying tasks.

**Method:**

Thirty participants (16 females and 14 male) performed a 10-min, 15kg load-carrying task with and without the exoskeleton in two non-consecutive days. Heart rate, and erector spinae, deltoid, biceps and brachioradialis muscle activity were recorded during the carrying tasks. In addition, erector spinae electromyography during an isometric hold test and discomfort ratings were measured before and after the task.

**Results:**

While without the exoskeleton upper limb muscle activity increased or remained constant during the carrying task and showing high peak activation for both males and females, a significant activity reduction was observed with the exoskeleton. Low back peak activation, heart rate and discomfort were lower with than without the exoskeleton. In males muscle activation was significantly asymmetric without the exoskeleton and more symmetric with the exoskeleton.

**Conclusion:**

The tested passive exoskeleton appears to alleviate the physical workload and impact of carrying heavy loads on the upper limbs and lower back for both males and females.

## Introduction

Carrying or moving heavy loads is commonly observed in both manufacturing and service industries. Workers most frequently exposed to carrying or moving heavy loads include personnel in healthcare, sales, agriculture, plant and machine operations [1], delivery, construction and mining [2], soldiers [3], among others. In the European Union, 32% of workers have reported that their work requires carrying and moving heavy loads at least one quarter of their workday, while in the US this statistic goes over 40% [1]. Carrying or moving heavy loads has been associated with several musculoskeletal disorders [4] including leg pain [5], hip and knee disorders [6], back pain [7, 8], chronic low back pain [9], lumbar spine disorders [10, 11], and neck and upper limb pain [7, 12]. The Bureau of Labor Statistics (BLS) reports that musculoskeletal injuries resulting in days away from work affect 38% of laborer and freight, stock and material movers, and 52% of nursing assistant [13].

Several ergonomic strategies have been proposed to reduce the risk of musculoskeletal disorders resulting from carrying and moving loads at work. These include automation of the carrying task [14], changing work practices and/or using equipment such as carts and cranes [15]. However, these strategies are not implementable for many occupational settings due to carrying task characteristics (e.g., object shape and prehension requirement), workplace design (space and configuration) and economic constraints, since automation is strongly cost and task compatibility dependent (e.g., complexity). Recently with advances in technology, new tools such as exoskeletons have been developed as an alternative to automation addressing physical burdens. Hence, their potential to mitigate work-related risks factors associated with musculoskeletal disorders have been explored [16, 17]. Some studies suggested that the aim of their use in occupational activities is to increase load handling capacity and endurance of workers in various industries [18].

Exoskeletons are wearable devices commonly classified as “active” or “passive”. Passive exoskeletons use only springs or elastic components to provide support to the user’s motion, while active exoskeletons are motorized. In occupational settings, passive exoskeletons have recently received more attention due to their commercial availability [19] and their “cost effectiveness and ease of implementation” [20]. Most passive exoskeletons have been designed to support the back in tasks requiring lifting and trunk flexion, the upper limbs when working with arms elevated, or the lower limbs during standing work [17]. From a systematic review, de Looze et al. [16] revealed that most passive industrial exoskeletons have been evaluated during lifting/lowering activities and/or with a small sample size (<15 participants). Many jobs that involve manual material handling require a combination of different tasks apart from lifting, such as carrying, moving and walking [21]. While the impact of passive exoskeletons in carrying and moving heavy loads has only recently received some attention, most studies focus on subjective discomfort and performance [21, 22] and less on objective measures, as reviewed by [23]. Moreover, the contribution of exoskeletons to muscle load reduction appears to be task and device specific [20].

Several studies have evaluated the effectiveness of exoskeletons through physiological outcomes such as muscle activity quantified by the amplitude of the root-mean-square of electromyographic signals (RMS EMG) see [16, 17, 24]. Fewer studies have estimated changes in muscle fatigue based on time variations of the median or mean frequency of the EMG power spectrum [25–27], or heart rate [28, 29]. Furthermore, other measures such as performance (e.g., time-to-task completion, maximal number of lifts, endurance time), subjective evaluations of discomfort and user’s satisfaction have been also considered when evaluating exoskeletons [29, 30]. Overall, several systematic reviews agree that passive exoskeletons may have the potential to reduce the influence of factors associated with work-related musculoskeletal disorders [16–18]. However, studies evaluating passive industrial exoskeletons with large sample sizes [16, 24] and long durations of exposure with tasks comparable to real work situations [30] are still needed. In addition, considering task characteristics, the specific design purpose of each exoskeleton [31], and sex differences are also necessary.

The aim of this study was to evaluate the effect of using a passive exoskeleton (Auxivo CarrySuit) on muscular activity of the lower back and upper limb, heart rate, discomfort, and satisfaction during a carrying and moving loads task considering the influence of sex. The study was designed to address the following questions:

- Does wearing the CarrySuit attenuate erector spinae (ES) muscle fatigue, heart rate and body area discomfort when compared to not wearing the exoskeleton during 10 min of carrying and moving a 15kg load?
- Does deltoid, biceps and brachioradialis muscular activity change over time during the carrying task with and without the exoskeleton?
- Is sex an important factor to consider in the present analysis?
- Do users prefer to perform the carrying and moving load task with than without the CarrySuit?

## Methods

### Participants

Thirty young healthy adults (16 females, 14 males) volunteered to participate in the study. All participants reported to be free from current or recent musculoskeletal pain or symptoms, pregnancy and/or any neurological or physical condition that could prevent them to perform a carrying task or to wear an exoskeleton. On average, the participants’ anthropometric characteristics were 21.19 **±** 1.6 years of age, 160.16 **±** 4.2 cm stature, and 56.58 **±** 7.24 kg weight for females, and 20.93 **±** 1.49 years of age, 173.39 **±** 6.01 cm stature, and 74.55 **±** 8.8 kg weight for males. Only three participants reported to be left-handed, and none had any previous experience with exoskeletons. The study was approved by the Ethics Committee of the Universidad San Francisco de Quito (#2021-145M) and complied with the tenets of the Declaration of Helsinki. All participants signed a written informed consent prior to any data collection.

### Exoskeleton for load carrying

**Fig 1:**
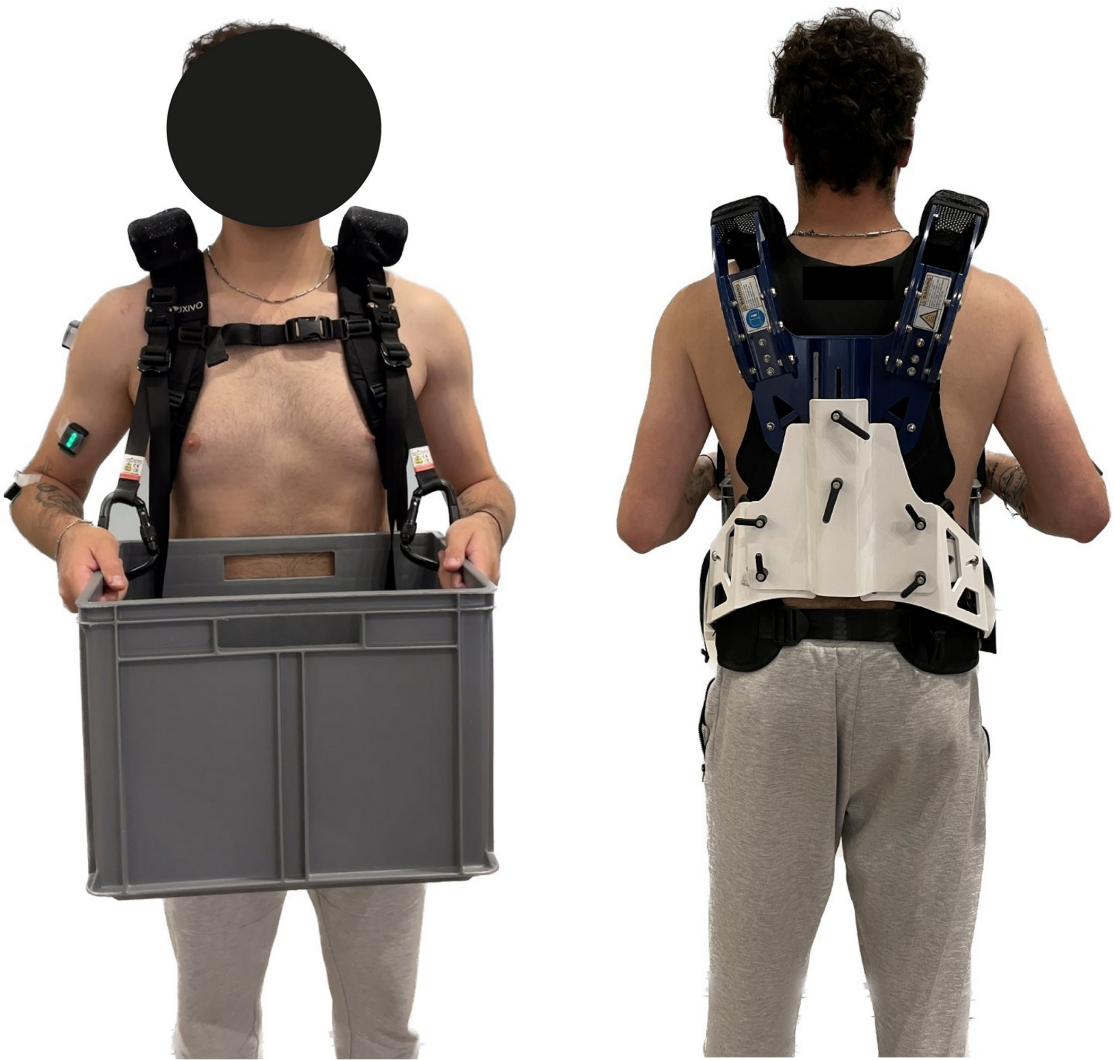
Participant wearing Auxivo CarrySuit and EMG sensors.

The Auxivo CarrySuit^Ⓡ^ v1.0, commercially released in 2021 by Auxivo AG (Schwerzenbach, Switzerland), was used in this study. This exoskeleton is an upper-body passive exoskeleton stated to reduce the load on the upper limbs and back when carrying and moving heavy loads [32]. The rigid exoskeleton frame extends from the hip to the shoulders in the posterior trunk area of the user. The frame is adjustable in width and height to conform to the anthropometry of the user and thus, connects to the body through hips at iliac crest level, chest, and shoulders via padded textile straps similarly to a backpack, as illustrated in Fig 1. External loads can be connected to the frame with carabiners and adapters. The exoskeleton weights 5.6 kg and was designed to support a maximum payload of 50 kg [32].

### Experimental design

The present study followed a crossover design, where all participants performed a treatment day and a control day assigned in a random order in two nonconsecutive days in a laboratory setting. Thus, participants served as their own controls. During the treatment day the carrying task was performed with the exoskeleton, while on the control day the same task was performed without any assistive device. The task consisted of repeating for 10 min the following sequence: carrying a 15kg box from one table to another separated by 3 meters (distance between pick up and drop off locations), putting the box for 1 second on the other table, and carrying it back to the first table. This is considered one carrying lap. The participants were instructed to perform as many laps as possible during that period, but at a comfortable and constant pace. On average 61.03 **±** 15.8 laps were performed with the exoskeleton and 63.7 **±** 14.9 without it. This minor difference was not significant. The height of both tables was adjusted so that the box handles were at elbows height to minimize forward bending. All participants were required to avoid strenuous physical activities such as weightlifting the day before each experimental day. The experimental days were separated by a minimum of 2 and a maximum of 5 days.

### Instrumentation and outcome measurements

#### EMG

The left and right ES activity was recorded and sampled at a frequency of 1926 Hz by two Trigno EMGs sensors (Delsys Inc, Boston, MA) placed at L3 height and about 3cm left and right from the spinous process. The deltoid medial, biceps, and brachioradialis muscle activity was recorded and sampled at 1260 Hz with Avanti EMGs sensors (Delsys Inc, Boston, MA) placed on the dominant arm at the greatest bulge of each muscle following international recommendations [33, 34]. All sensors were bipolar, surface, and wireless with a 10 mm interelectrode distance. Before placing the sensors, the skin was shaved, if necessary, and cleaned using abrasive gel (Skin Prep Gel, Nuprep®, Aurora, USA). The sensors placements were marked with a temporary “bodymarker” to ensure an exact re-positioning on the second experimental day.

#### Heart rate

Heart beats per minute (bpm) was quantified before and during the carrying task, from a previously validated [35] real time Polar H10 heart rate Sensor System (Polar ©, Findland) strapped to the chest. Resting heart rate was measured at the beginning of each experimental session after sitting in an armchair and resting for 5 minutes. The lowest value was recorded. Heart rate was also recorded during the 10-min of the carrying task and the mean value was computed.

#### Subjective evaluation

Discomfort perception and satisfaction of the exoskeleton was evaluated with a questionnaire. The first section, adapted from the Nordic questionnaire [36], included perception of discomfort at the neck, shoulders, upper back, arms, lower back, and hand/wrists, as in our previous studies [37, 38]. A 0–10 cm visual analog scale was placed next to each of these body areas, where “no discomfort” (0) corresponded to the far left of the scale and “extreme discomfort” (10) to the far right. Vertical marks over the scales indicated the perceived level of discomfort. An additional question about overall perception of fatigue was also included using an identical rating scale. This section of the questionnaire was filled before and after the carrying task on each experimental day. The second section of the questionnaire, which was filled only after the last experimental day, included four questions to rate: how easy was to perform the task with the exoskeleton, how comfortable was to perform the task with the exoskeleton, whether performance of the task was preferred with or without the exoskeleton and whether the exoskeleton would be recommended for carrying tasks.

### Experimental protocol

Upon participants’ arrival on the first experimental day, the study was explained, the exoskeleton was introduced, then the inform consent was signed and demographic data were collected. For both experimental days, the subjective evaluation and resting heart rate were recorded first and then EMG electrodes were placed on the selected muscles. Afterwards, ES EMGs were recorded during an isometric test requiring the participants to maintain for 30 seconds a 45° trunk flexion while standing with flat back, knees straight, arms hanging relax and holding a 5kg weight with both hands. Two warm-up trials separated by a 5 min seated rest preceded this test before recording. The same posture was ensured across trials and days for each participant using visual feedback from an inertial measurement unit (IMU) sensor located below C7. The magnitude of the imposed inclination was indicated by a horizontal line on a computer screen and the vertical displacement of a cursor moving horizontally as a function of time corresponded to the torso inclination magnitude, which corresponded to a simple tracking task. This isometric test, performed before and after the carrying task, was used to evaluate lower back muscle fatigue, as in other exoskeleton studies [25, 39]. During the treatment day, the exoskeleton was fit to each participant according to the manufacturer guidelines and the participant’s comfort. To get familiarized with the task, a 1 min warm-up session of carrying and moving a 5kg weight was performed with or without the exoskeleton depending upon the condition tested on the day. Subsequently, the 10 min carrying task was performed, during which EMG data were recorded every two minutes (T0, T1, T2, T3, T4, T5) for one carrying lap and heart rate recorded continuously. At the end of the carrying task the isometric hold test was performed again as well as the subjective evaluations of discomfort and fatigue. The user’s satisfaction questionnaire was filled after completion of the carrying task only on the second experimental day, regardless of condition order. Overall, each experimental day per participant took ~1 hour. The dependent variables consisted of EMG median frequency slope, RMS EMG 10^th^, 50^th^ and 90^th^ percentile, mean beats per min, and discomfort scores.

### EMG data processing

All data were processed in MATLAB (The MathWorks Inc., Natick, MA, USA) using a custom script to apply a set of EMG signal processes [see 25, 39–41]. The ES EMG acquired during the isometric tests were first detrended and then filtered with a 4^th^-order Butterworth bandpass filter (10-400Hz) using the zero-phase digital function. This signal was then divided into none-overlapping 1-s epochs. For each epoch, Welch’s power spectral density estimate was applied with a Hanning window, and the median frequency computed. The polynomial curve fitting function was used to find the best least square linear fit to the successive median frequencies and determine the slope of this line for data recorded before and after the carrying task.

For the EMGs recorded during the carrying task the signals were detrended and then filtered with a 4^th^-order Butterworth bandpass filter (30-300Hz) using the zero-phase digital function. A Fast Fourier Transform function converted each signal into the frequency domain to visually evaluate the quality of the EMG data. The signals were then full wave rectified using the absolute value of the filtered data to calculate the reference value for normalization purposes. The RMS EMGs were also computed with a 250 ms moving window with 50% overlap. Then for each experimental day the data were normalized to the submaximal voluntary contraction performed during the first isometric test (described above) for the ES and to the mean activation value obtained during the first 30 seconds of the carrying task (T0) for the upper limb muscles. Both methods are common in EMG studies, as reviewed by Burden A. [42]. The RMS EMG 10^th^, 50^th^ and 90^th^ percentiles (P_10_, P_50_, P_90_) were calculated to describe the distribution of the muscle activity during the task [26, 43–46]. The 10^th^, 50^th^ and 90^th^ percentiles represent respectively the static, mean and peak muscular activity [46, 47]. Finally, for P_90_ of the upper limbs the slopes (over time) were calculated through MATLAB’s polynomial curve fitting function.

### Statistical data analysis

The following dependent variables: median frequency EMG, RMS EMG, and heart rate, were analyzed with mixed models with a variance-components covariance structure with a residual maximum likelihood estimation. These data fulfilled the normality assumption according to the Kolmogorov-Smirnov test. For the statistical models, participants were considered as random effects and measurement time, sex, and condition (with [EXO] or without the exoskeleton [NOEXO]) as fixed effects. However, for the upper limbs EMG data the factor “condition” was not included in one model since normalization was performed to the mean muscle activation recorded at T0 on the corresponding day, thus, two separate models were tested for each condition. However, an additional model was used for the slopes of P_90_ RMS EMG that included condition and sex. For the ES data an additional model was used to compare right versus left RMS EMG per condition, including side as a fixed effect and excluding the three left-handed participants only for this analysis. Post-hoc analysis consisted of least square means differences with Tukey-Kramer adjustment of p-values due to multiple comparisons performed only with the significant factors of each model. The results present the relevant means (M) and standard errors (SE). All statistical analysis was performed in SAS Studio (SAS Institute Inc.) and the significance level was set to α = .05. Partial eta-squared pseudo-effect size (η_p_^2^) was calculated using Tippey & Longnecker [48] method for mixed models in SAS Studio. Cohen benchmarks defining small (η_p_^2^ = .01), medium (η_p_^2^ = .06) and large effects (η_p_^2^ = .14) were used for interpretation of η_p_^2^ [49]. Discomfort data, which did not fulfill the normality assumption, was analyzed with Friedman nonparametric two-way analysis of variance by ranks test.

## Results

### Isometric test erector spinae EMG

Neither the effects of time, *F*(1,71) = .21, p = .65, η_p_^2^ = .0002, condition, *F*(1,71) = .02, p = .88, η_p_^2^ = .00002, and sex *F*(1,71) = .01, p = .92 η_p_^2^ = .0001, nor their three-way interaction *F*(2,71) = .45, p = .64, η_p_^2^ = .0003 were significant for the slope of the right ES median frequency. Similarly, for the slope of the left ES median frequency the effects of time, *F*(1,73) = .13, p = .72, η_p_^2^ = .001, condition, *F*(1,73) = 2.37, p = .12, η_p_^2^ = .02, and sex *F*(1,73) = .69, p = .40 η_p_^2^ = .006, as well as their three-way interaction *F*(2,73) = 1.68, p = .19, η_p_^2^ = .03 were not significant. In addition, changes in the y-intercept of median frequency regression lines were not significant for either of the ES sides.

### Carrying task erector spinae EMG

**Fig 2:**
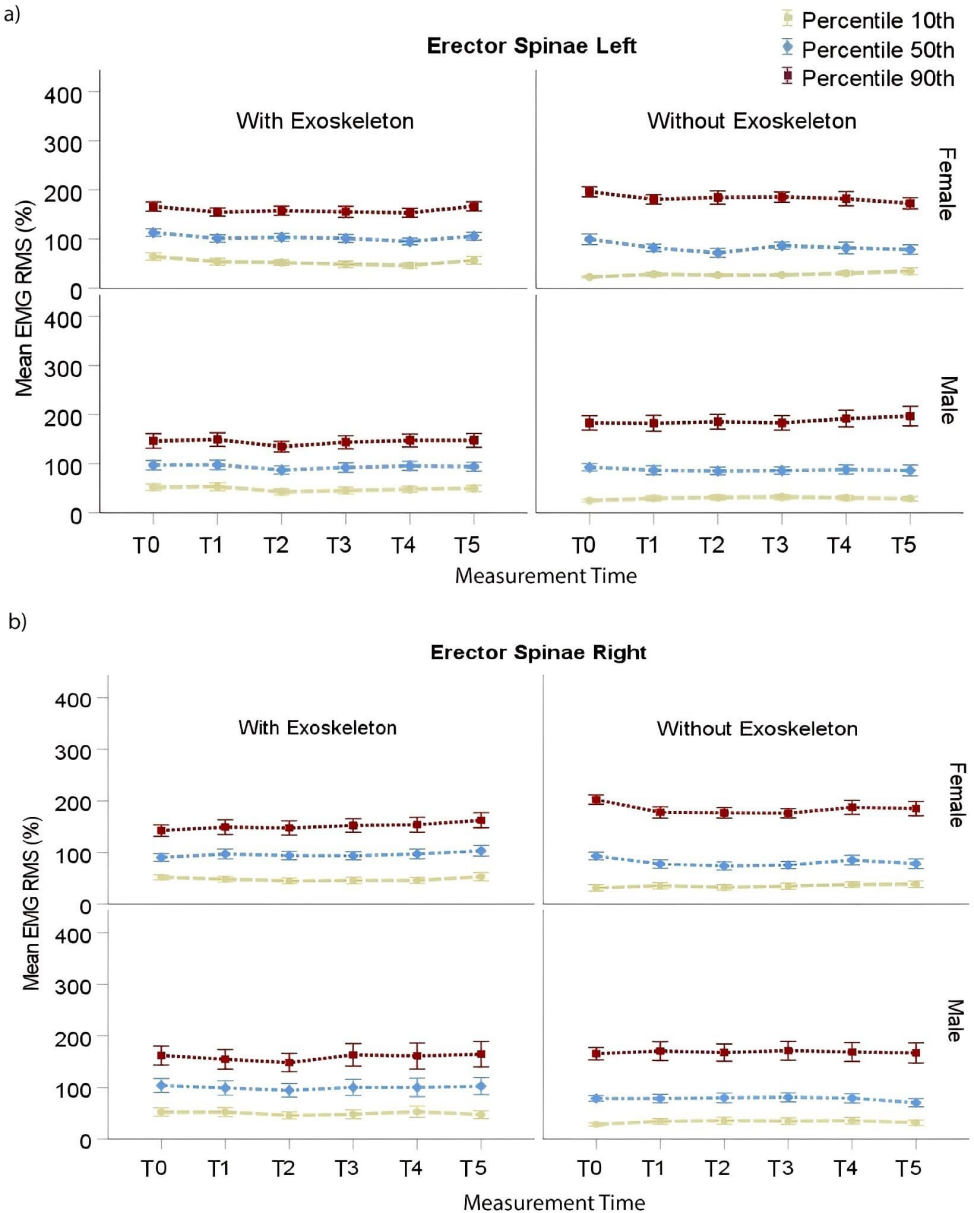
Erector Spinae right (a) and left (b) RMS EMG 10^th^, 50^th^ and 90^th^ percentiles for males and females. Vertical bars indicate standard errors.

RMS EMG P_10_, P_50_, and P_90_ of the ES are presented in Table 1 for the right side and Table 2 for the left side. Post-hoc comparisons for the right ES P_10_ showed significantly higher values (p < .0001) in the EXO (M = 51.15%, SE = 3.75%) than NOEXO (M = 33.05%, SE = 3.74%) condition regardless of time and sex. Similarly, P_50_ in the EXO condition (M = 100.57%, SE = 5.73%) was significantly higher (p < .0001) than in NOEXO (M = 76.47%, SE = 5.72%) regardless of time and sex. However, P_90_ in the EXO condition (M = 150.62%, SE = 12.66%) was significantly lower (p = .0008) than in NOEXO (M = 175.43%, SE = 12.75%) regardless of time for females but not for males. For the left ES P_10_ was significantly higher (p < .0001) for the EXO condition regardless of time when compared to NOEXO, for males (EXO: M = 48.05%, SE = 4.63%; NOEXO: M = 29.56%, SE = 4.55%) and females (EXO: M = 53.55%, SE = 4.42%; NOEXO: M = 23.27%, SE = 4.51%). However, for P_50_ the values where significantly higher for the EXO condition (p < .0001) only for females (EXO: M = 102.54%, SE = 6.51%; NOEXO: M = 79.68%, SE = 6.67%). For P_90_, peak activity was significantly lower (p < .0001) in the EXO than NOEXO condition regardless of time for males (EXO: M = 142.25%, SE = 10.64%; NOEXO: M = 187.24%, SE = 10.49%) and females (EXO: M = 158.57%, SE = 10.19%; NOEXO: M = 182.57%, SE = 10.38%). These results are illustrated in Fig 2.

**Table 1:** Right ES RMS EMG percentiles.

	10^th^ percentile (P_10_)			50^th^ percentile (P_50_)			90^th^ percentile (P_90_)		
Effect	F value	p value	η_p_^2^	F value	p value	η_p_^2^	F value	p value	η_p_^2^
Condition	90.47	< .0001*	0.004	62.04	< .0001*	0.004	11.5	0.0008*	0.005
Time	0.25	0.94	0.20	0.29	0.91	0.15	0.33	0.89	0.03
Sex	0.001	0.97	0	0.11	0.74	0	0.03	0.87	0
Condition x Time	0.74	0.59	0.01	0.51	0.77	0.007	0.34	0.89	0.005
Condition x Sex	0.25	0.61	0.007	0.02	0.89	0.005	4.53	0.03*	0.01
Sex x Time	0.52	0.76	0.007	0.22	0.95	0.003	0.16	0.97	0.002
Condition x Sex x Time	0.15	0.99	0.003	0.5	0.81	0.009	1.25	0.28	0.02

Note. Bold font and * indicate significance at α = 0.05

**Table 2:** Left erector spinae EMG RMS percentiles.

	10^th^ percentile (P_10_)			50^th^ percentile (P_50_)			90^th^ percentile (P_90_)		
Effect	F value	p value	η_p_^2^	F value	p value	η_p_^2^	F value	p value	η_p_^2^
Condition	253.2	< .0001*	0.01	32.87	< .0001*	0.034	104.4	< .0001*	0.01
Time	0.73	0.60	0.42	2.38	0.03*	0.086	0.45	0.82	0.23
Sex	0	0.95	0	0	0.97	0	0.16	0.69	0
Condition x Time	3.39	0.005*	0.05	0.29	0.92	0.004	0.17	0.97	0
Condition x Sex	14.82	0.0001*	0.04	10.57	0.001*	0.03	9.67	0.002*	0.03
Sex x Time	0.67	0.65	0.01	0.73	0.60	0.011	0.83	0.53	0.01
Condition x Sex x Time	3.28	0.004*	0.05	2.05	0.06	0.035	1.99	0.07	0.03

Note. Bold font and * indicate significance at α = 0.05

Right versus left ES RMS EMG comparisons per condition are presented in Table 3. Post hoc comparisons showed a significantly higher (p = .03) P_10_ activity on the left ES (M = 51.13%, SE = 4.32%) than the right (M = 48.01%, SE = 4.34%) in the EXO condition over time, for both sexes. Oppositely, P_10_ activity was lower (p = .0007) for the left ES (M = 30.88%, SE = 3.36%) than the right (M = 35.21%, SE = 3.37%) in the NOEXO condition. And, P_50_ activity was higher (p = .001) for the left ES (M = 87.39%, SE = 5.18%) than the right (M = 80.04%, SE = 5.21%) side in the NOEXO condition over time for both sexes; while no differences were found in the EXO condition. For P_90_ in the NOEXO condition, activity was higher (p = .0002) for the left ES (M = 187.24%, SE = 12.30%) than the right (M = 165.84%, SE = 12.44%) side only for males over time, while no differences were found in the EXO condition.

**Table 3:** Right versus left erector spinae RMS EMG percentiles per condition.

		10^th^ percentile (P_10_)			50^th^ percentile (P_50_)			90^th^ percentile (P_90_)		
Condition	Effect	F value	p value	η_p_^2^	F value	p value	η_p_^2^	F value	p value	η_p_^2^
With Exoskeleton (EXO)	Sex	0.01	0.91	.0002	0.07	0.79	0.0005	0.08	0.78	0.0009
	Time	2.92	0.01*	0.04	0.43	0.82	0.007	0.45	0.81	0.006
	Side	4.36	0.03*	0.01	0.45	0.50	0.0009	1.45	0.22	0.005
	Sex x Side	2.45	**0.11**	0.007	1.32	0.25	0.004	2.76	0.09	0.008
	Time x Side	1.09	0.36	0.01	0.72	0.61	0.01	0.31	0.90	0.008
	Sex x Time	1.31	0.25	0.01	0.10	0.99	0.001	0.17	0.97	0.003
	Sex x Time x Side	0.43	0.83	0.006	0.89	0.48	0.01	0.63	0.67	0.007
Without Exoskeleton (NOEXO)	Sex	0.09	0.76	0.0008	0.01	0.91	0.00003	0.17	0.67	0.00036
	Time	3.24	0.007*	0.04	2.76	0.01*	0.03	0.51	0.76	0.007
	Side	11.65	0.0007*	0.03	10.78	0.001*	0.03	7.75	0.005*	0.02
	Sex x Side	0.29	0.58	0.001	0.53	0.47	0.001	9.56	0.002*	0.02
	Time x Side	0.12	0.98	0.001	0.58	0.71	0.006	0.05	0.99	0.001
	Sex x Time	1.66	0.14	0.02	1.55	0.17	0.02	1.08	0.37	0.01
	Sex x Time x Side	0.14	0.98	0.002	0.47	0.79	0.006	0.76	0.57	0.01

Note. Bold font and * indicate significant values, α = 0.05

### Carrying task RMS EMG upper limb muscles

#### Deltoid

RMS EMG P_10_, P_50_, and P_90_ in both tested conditions are presented in Table 4. In the EXO condition, post hoc comparisons showed a significant decrease in P_10_ from T0 (*M* = 72.12%, SE = 5.39%) to T1(*M* = 59.38%, SE = 5.39%; adj p = .01), through T5(*M* = 59.21%, SE = 5.51%; adj p = .01) regardless of sex. A similar decrease in P_50_ from T0 (*M* = 118.96%, SE = 6.94%) to T1(*M* = 92.44%, SE = 6.94%; adj p = .01), through T5 (*M* = 86.74%, SE = 7.31%; adj p = .001) was observed regardless of sex. Also, a decrease in P_90_ from T0 (*M* = 215.91%, SE = 13.62%) to T1(*M* = 168.16%, SE = 13.62%; adj p = .0006), through T5 (*M* = 149.53%, SE = 14.39%; adj p = .001) was found regardless of sex.

**Table 4:** Deltoid RMS EMG percentiles.

		10^th^ percentile (P_10_)			50^th^ percentile (P_50_)			90^th^ percentile (P_90_)		
Condition	Effect	F value	p value	η_p_^2^	F value	p value	η_p_^2^	F value	p value	η_p_^2^
With Exoskeleton (EXO)	Sex	3.8	0.05	0.02	3.31	0.07	0.02	2.55	0.11	0.01
	Time	4.39	0.001*	0.11	5.43	0.0002*	0.13	5.44	0.0002*	0.13
	Sex x Time	0.42	0.83	0.01	1.17	0.33	0.03	1.33	0.26	0.04
Without Exoskeleton (NOEXO)	Sex	1.98	0.16	0.01	1.53	0.22	0.008	0.58	0.45	0.003
	Time	1.88	0.10	0.05	4	0.002*	0.11	5.89	< .0001*	0.14
	Sex x Time	1.06	0.38	0.03	1.94	0.09	0.05	1.71	0.14	0.05

Note. Bold font and * indicate significant values, α = 0.05

**Fig 3:**
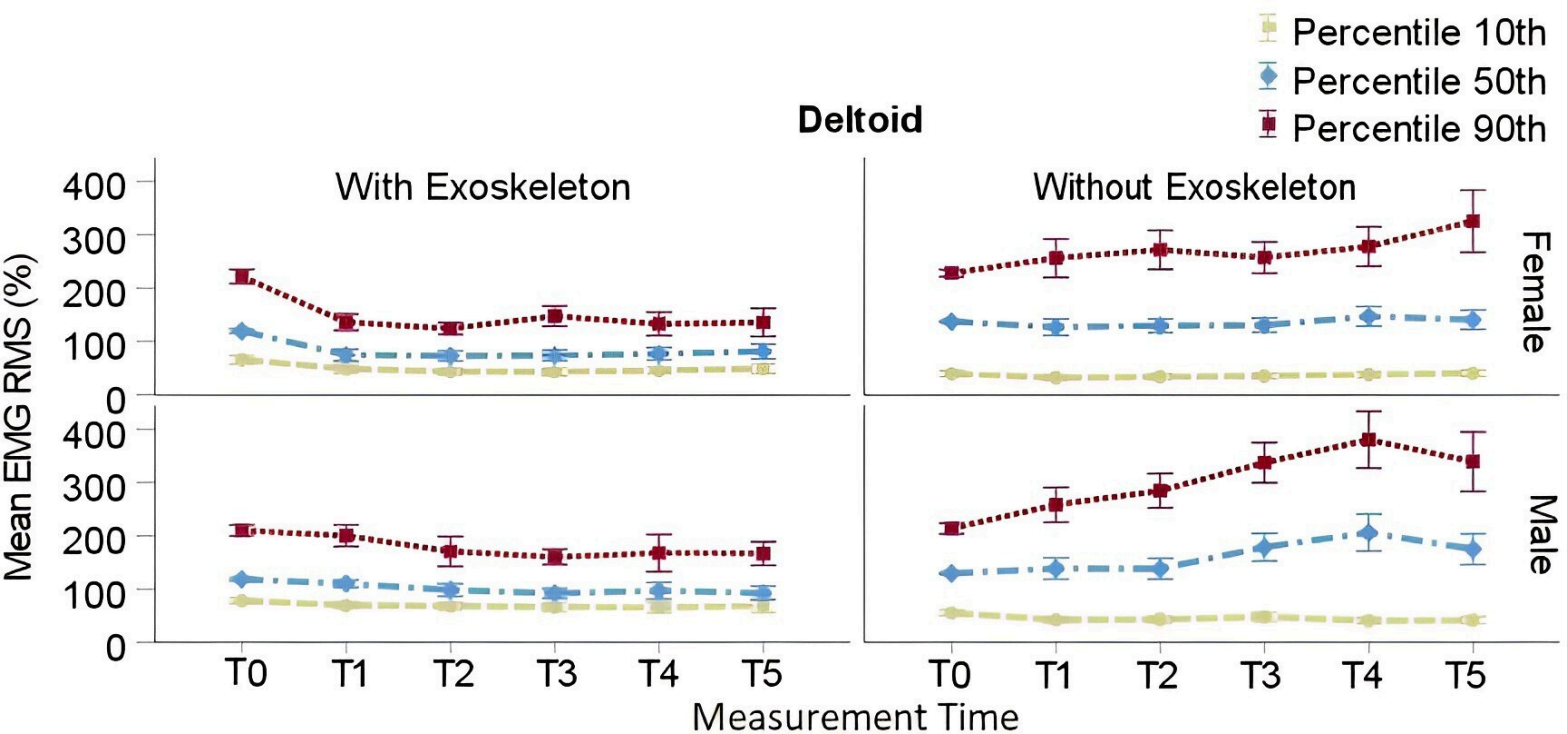
Deltoid RMS EMG 10^th^, 50^th^ and 90^th^ percentiles for males and females. Vertical bars indicate standard errors.

In the NOEXO condition post hoc comparisons showed no significant differences through time in P_10_ regardless of sex. When compared to T0 (*M* = 133.41%, SE = 12.96%; adj p = .01), a significant increase in P_50_ was found at T1 (*M* = 131.21%, SE = 13.34% adj p = .01), T2 (*M* = 131.04%, SE = 13.25% adj p = .01) and T4 (*M* = 175.67%, SE = 13.15%), regardless of sex. A significant increase in P_90_ from T0 (*M* = 220.94%, SE = 26.71%) to T2 (*M* = 272.58%, SE = 27.27%, adj p = .04) through T5 (*M* = 331.62%, SE = 27.07%, adj p = < .0001) was found regardless of sex. These results are illustrated in Fig 3.

The slopes analysis for P_90_ showed a main effect of condition *F*(1,19) = 15.84, p = .0008, η_p_^2^ = .24. However, the effects of sex, *F*(1,19) = 1.21, p = .28, ηp2 = .02, and time*sex interaction, *F*(1,19) = .33, p = .57, ηp2 = .006, were not significant. Post hoc comparisons showed a significant difference between the slopes in both conditions regardless of sex. The slope was found positive in the NOEXO (*M* = 22.87, SE = 5.73) and negative in the EXO (*M* = -11.40, SE = 6.42) condition, respectively.

#### Biceps

RMS EMG P_10_, P_50_, and P_90_ in both tested conditions are presented in Table 5. In the EXO condition post-hoc comparisons showed a significant decrease in P_10_ from T0 (*M* = 55.21%, SE = 4.17%) to T1 (*M* = 41.77%, SE = 4.24%; adj p = .003) through T5 (*M* = 32.22%, SE = 4.27%; adj p = < .0001) regardless of sex. A significant decrease in P_50_ from T0 (*M* = 123.79%, SE = 8.53%) to T1 (*M* = 76.89%, SE = 8.70%; adj p = < .0001) through T5 (*M* = 87.45%, SE = 8.89%; adj p = .003) was found for females, and from T0 (*M* = 123.21%, SE = 8.83%) to T3 (*M* = 82.05%, SE = 9.52%; adj p = .001) and T5 (*M* = 82.99%, SE = 9.03%; adj p = .0007) for males. A significant decrease in P_90_ from T0 (*M* = 228.18%, SE = 11.91%) to T1 (*M* = 181.83%, SE = 12.28%; adj p = .02) trough T5 (*M* = 163.71%, SE = 12.46%; adj p = .0004), was found regardless of sex.

**Table 5:** Biceps RMS EMG percentiles.

		10^th^ percentile (P_10_)			50^th^ percentile (P_50_)			90^th^ percentile (P_90_)		
Condition	Effect	F value	p value	η_p_^2^	F value	p value	η_p_^2^	F value	p value	η_p_^2^
With Exoskeleton (EXO)	Sex	0.6	0.44	0.003	1.61	0.20	0.009	0.19	0.66	0.001
	Time	12.04	<0.0001*	0.26	12.48	<0.0001*	0.26	6.89	<0.0001*	0.16
	Sex x Time	1.12	0.35	0.03	3.79	0.003*	0.1	1.25	0.28	0.03
Without Exoskeleton (NOEXO)	Sex	0.76	0.38	0.004	4	0.04*	0.02	6.25	0.01*	0.03
	Time	1.28	0.27	0.04	1.75	0.120	0.05	2.42	0.03*	0.06
	Sex x Time	1.06	0.38	0.03	2.84	0.01*	0.07	1.91	0.09	0.05

Note. Bold font and * indicate significant values, α = 0.05

**Fig 4:**
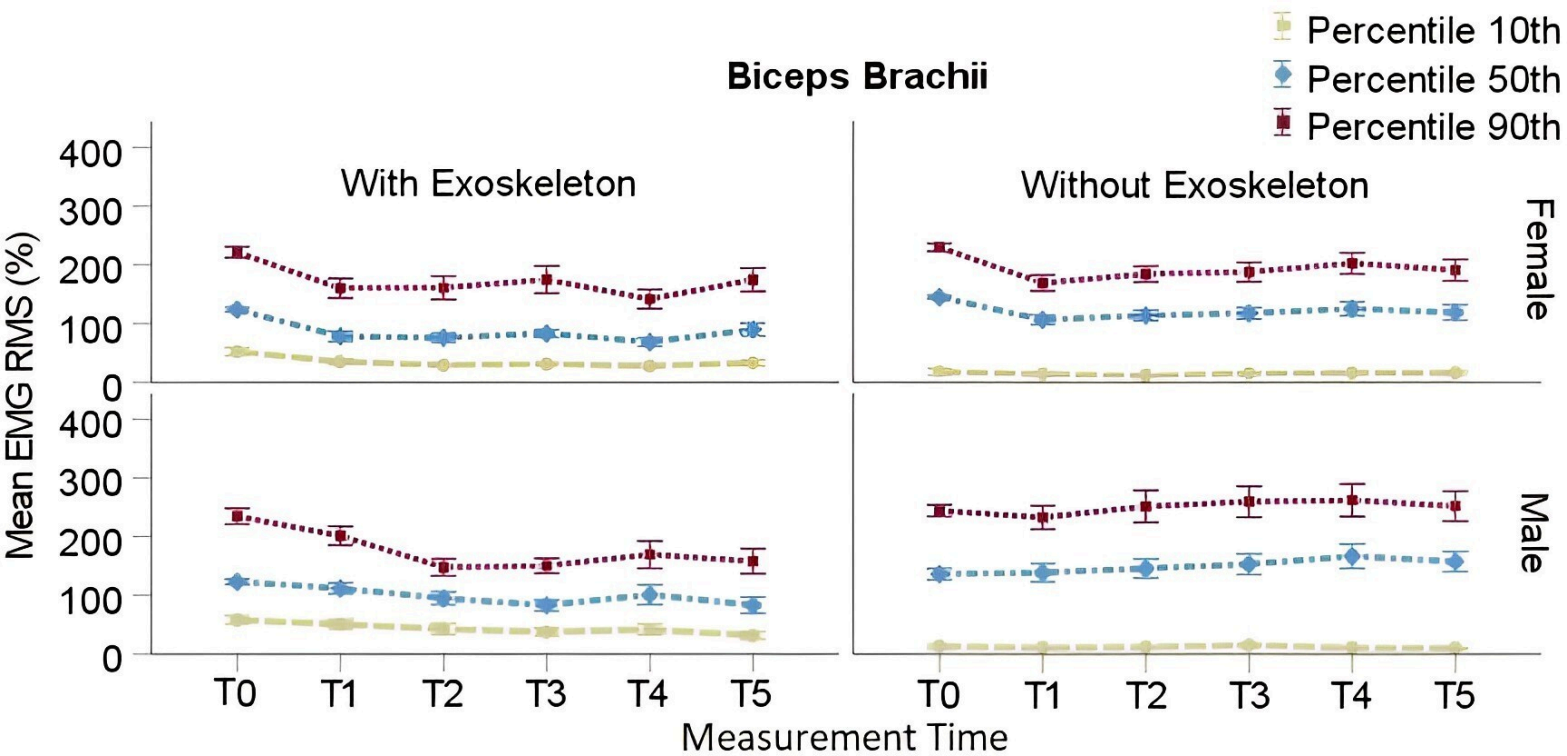
Biceps RMS EMG 10^th^, 50^th^ and 90^th^ percentiles for males and females. Vertical bars indicate standard errors.

In the NOEXO condition post hoc comparisons showed no significant differences as a function of time for P_10_ regardless of sex. A significant decrease in P_50_ from T0 (*M* = 145.13%, SE = 12.10%) to T1 (*M* = 106.54%, SE = 12.11%; adj p = .02) was found for females, but then P_50_ increased again since T2 through T5 is not significantly different from T0. Similarly for females P_90_ decreased significantly from T0 (*M* = 229.91%, SE = 17.91%) to T1 (*M* = 169.20%, SE = 17.92%; adj p = .003), but then the P_90_ increased again since T2 through T5 is not significantly different from T0. No significant changes were found for males for any percentile. These results are illustrated in Fig 4.

The slopes analysis for P_90_ showed a main effect of condition *F*(1,22) = 8.83, p = .007, η_p_^2^ = .14. However, the effects of sex, *F*(1,22) = .17, p = .68, ηp2 = .003, and time*sex interaction, *F*(1,22) = 1.17, p = .29, ηp2 = .02, were not significant. Post hoc comparisons showed a significant difference between the slopes of both conditions regardless of sex. The slope was found positive in the NOEXO (*M* = .62, SE = 2.85) and negative in the EXO (*M* = -11.86, SE = 3.08) condition, respectively.

#### Brachioradialis

RMS EMG P_10_, P_50_, and P_90_ in both tested conditions are presented in Table 6. In the EXO condition post hoc comparisons showed a significant decrease of P_10_ from T0 (*M* = 51.09%, SE = 6.31%) to T3 (*M* = 34.56%, SE = 6.73%; adj p = .05) through T5 (*M* = 36.14%, SE = 6.50%; adj p = .05) for males, and no changes for females. A decrease of P_50_ from T0 (*M* = 115.13%, SE = 9.12%) to T3 (*M* = 85.90%, SE = 10.29%; adj p = .05) through T5 (*M* = 76.24%, SE = 9.65%; adj p = .007) was found for males, and from T0 (*M* = 118.80%, SE = 8.81%) to T1 (*M* = 80.78%, SE = 9.01%; adj p = .003) through T5 (*M* = 86.49%, SE = 9.22%; adj p = .03) for females. A significant decrease of P_90_ from T0 (*M* = 233.79%, SE = 11.13%) to T2 (*M* = 165.53%, SE = 12.31%; adj p = .0002) through T5 (*M* = 159.55%, SE = 11.92%; adj p = < .0001) was found regardless of sex.

**Table 6:** Brachioradialis EMG RMS percentiles.

		10^th^ percentile (P_10_)			50^th^ percentile (P_50_)			90^th^ percentile (P_90_)		
Condition	Effect	F value	p value	η_p_^2^	F value	p value	η_p_^2^	F value	p value	η_p_^2^
With Exoskeleton	Sex	0.03	0.86	0.0001	0.59	0.44	0.003	0.42	0.51	0.002
	Time	3.33	0.007*	0.09	7.85	< .0001*	0.18	7.07	< .0001*	0.17
	Sex x Time	2.25	0.05*	0.06	1.94	0.09*	0.05	0.81	0.54	0.02
Without Exoskeleton	Sex	2.36	0.12	0.01	6.29	0.01*	0.03	3.17	0.07	0.02
	Time	2.58	0.02*	0.07	1.37	0.230	0.04	1.31	0.26	0.04
	Sex x Time	0.57	0.72	0.01	2.22	0.05	0.06	1.23	0.29	0.03

Note. Bold font and * indicate significant values, α = 0.05

**Fig 5:**
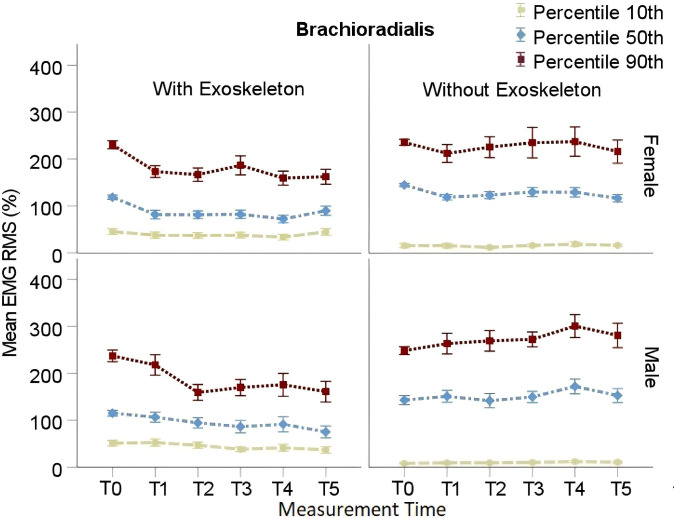
Brachioradialis RMS EMG percentile 10^th^, 50^th^ and 90^th^ for males and females. Vertical bars indicate standard errors.

In the NOEXO condition post hoc comparisons showed no significant differences as a function of time for P_10_ regardless of sex. Similarly, no significant changes were found for P_50_ and P_90_. These results are illustrated in Fig 5.

The slopes analysis for P_90_ showed a main effect of condition *F*(1,21) = 14.10, p = .001, η_p_^2^ = .22. However, the effects of sex, *F*(1,21) = .37, p = .54, ηp2 = .007, and time*sex interaction, *F*(1,21) = 1.37, p = .26, ηp2 = .03, were not significant. Post hoc comparisons showed a significant difference between the slopes of both conditions regardless of sex. the slope was found positive in the NOEXO (*M* = 3.76, SE = 3.38) and negative in the EXO (*M* = -13.04, SE = 3.68) condition, respectively.

### Heart rate

**Fig 6:**
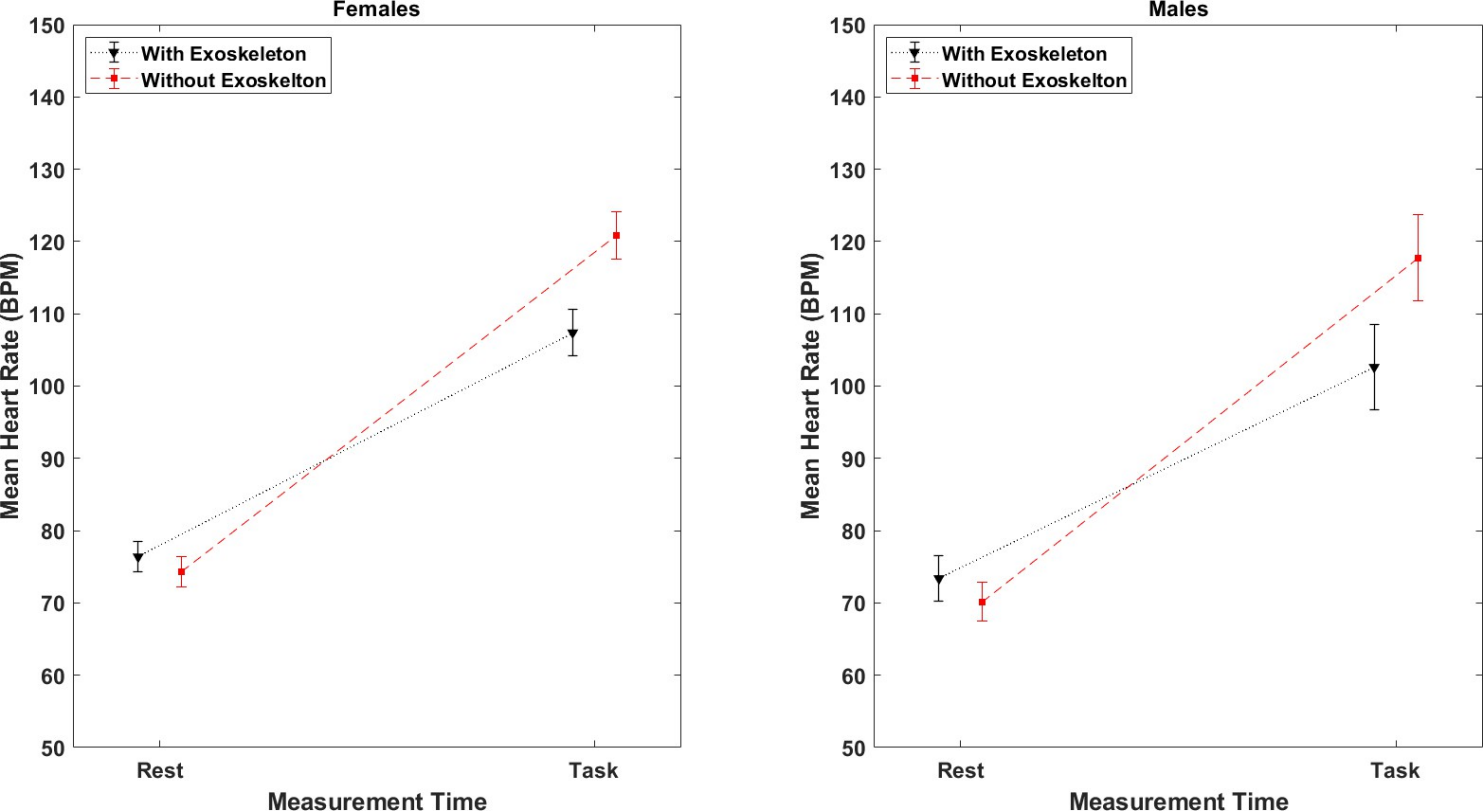
Mean heart rate comparisons for males and females. Vertical bars indicate standard errors.

A main effect of time, *F*(1,74) = 504.11, p = < .0001, η_p_^2^ = .82, condition, *F*(1,74) = 16.86, p = .0001, η_p_^2^ = .13, and a significant time*condition interaction, *F*(1,74) = 24.26, p = < .0001, η_p_^2^ = .18, were observed. However, the effect of sex was not significant, *F*(1,74) = .43, p = .51, η_p_^2^ = .004, as well as its two and three way interactions. Post hoc comparisons showed a significantly higher heart rate (all values in bpm) during the task when compared to the resting heart rate for both conditions. However, the increase of heart rate during the carrying task in the NOEXO condition (Females: *M* = 121.06, SE = 3.78; adj p = .002, Males: *M =* 119.99, SE = 4.06; adj p = .0002) was significantly higher than in the EXO condition (Females: *M* = 107.38, SE = 3.64, Males: *M* = 102.10, SE = 3.97). The resting heart rate between conditions was not significantly different for both females and males. These results are illustrated in Fig 6.

### Subjective evaluation of discomfort

Discomfort rating comparisons (Friedman test) are presented in Table 7. The left panel corresponds to the before vs. after the carrying task in each condition, respectively. These results show that discomfort in all body areas and overall fatigue were significantly higher in the NOEXO condition when compared to baseline values. However, in the EXO condition discomfort was significantly higher only for the neck, shoulders, upper back, and overall fatigue. The right panel in Table 6 corresponds to the difference between conditions (EXO and NOEXO), by computing the difference (Δ) between ratings reported after the carrying task minus the ratings reported before (baseline). These results show that the elbows/arms, hand/wrists discomfort, and overall fatigue were higher in the NOEXO than EXO condition.

**Table 7:** Discomfort ratings.

	Before vs After Carrying Task				With vs Without Exoskeleton	
	With Exoskeleton (EXO)		Without Exoskeleton (NOEXO)		Δ(After-Before)	
	F value	p value	F value	p value	F value	p value
Neck	12.43	0.001*	24.58	<0.0001*	0.04	0.85
Shoulders	39.43	<0.0001*	10.85	0.003*	1.4	0.25
Upper Back	31.82	<0.0001*	54.59	<0.0001*	3.22	0.08
Elbows/Arms	0	1	36.25	<0.0001*	16	0.0004*
Lower Back	3.46	0.07	10.63	0.003*	1.53	0.23
Hands/Wrists	0.06	0.81	16.09	0.0004*	11.15	0.002*
Overall Fatigue	12.71	0.001*	51.56	<0.0001*	16.31	0.0004*

Note. F value indicates Friedman’s F. Bold font and * indicate significant values, α = 0.05

In the second section of the questionnaire, 18 of the 30 participants scored above 7/10 on the easiness to perform the task with the exoskeleton. For the comfortability question, 16 of the 30 participants scored above 7/10. In terms of preference, 25 of the 30 participants preferred to carry with than without the exoskeleton. Finally, 26 of the 30 participants will recommend the use of this exoskeleton for carrying tasks.

## Discussion

The present study investigated the effect of a passive exoskeleton CarrySuit^Ⓡ^, on muscle activity, fatigue, heart rate and discomfort during a 10-min carrying and moving a load task. Overall, the exoskeleton favored the reduction of activity in upper arm muscles, while without the exoskeleton, upper limb activity increased or remained constant during the carrying task and presented a higher difference in peak activation (P_90_) relative to the mean activation (P_50_). Similarly, for the ES muscles, peak activation was higher without than with the exoskeleton. Heart rate and perceived discomfort were also lower with than without the exoskeleton, which concur with the other objective measures.

### Low back muscle activity and fatigue

Using the exoskeleton to carry a load led to a reduction of the peak EMG activity (P_90_) of the ES on both sides for females and only on the left side for males, when compared to not wearing the exoskeleton. However, the static (P_10_) and mean (P_50_) activity were generally higher with than without the exoskeleton. Also, a significant ES load asymmetry was observed for the mean (P_50_) and peak (P_90_) EMG activity in the NOEXO condition, for males but not females. ES activity was more symmetric in the EXO condition for males and in both conditions for females.

**Fig 7:**
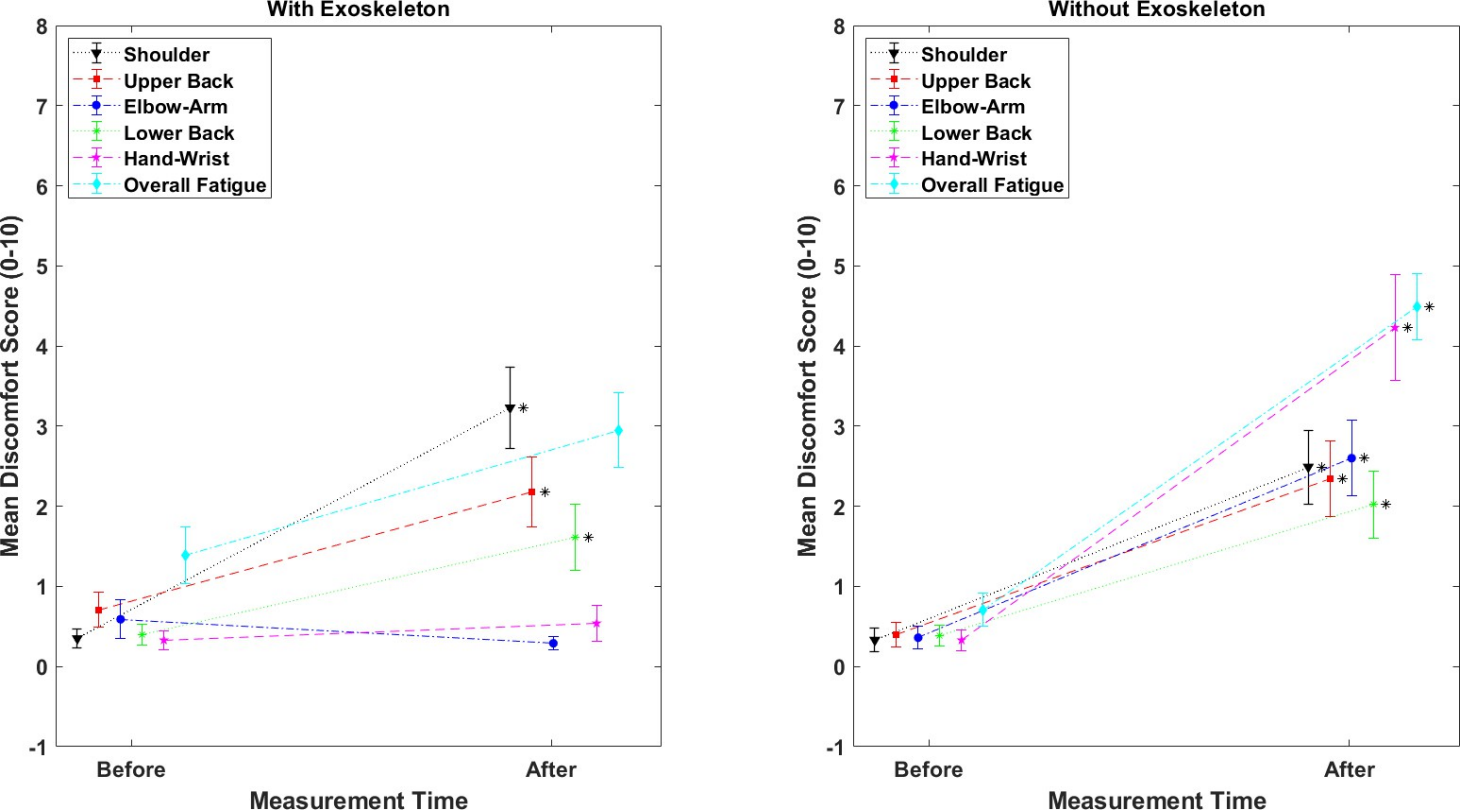
Shoulder, elbow/arms, hands/wrists and upper back discomfort, and overall fatigue ratings (0–10). Vertical bars indicate standard errors.

Taken together these results illustrate an example of tradeoff in the utilization of an exoskeleton. From a biomechanical point of view, the increase in static and mean ES activity is most likely associated with the increase in total load due to the added weight of the exoskeleton, which represents about 30% of the box weight. However, as the exoskeleton is designed to redistribute forces by contact with the hip, the lower peak activity (P_90_) resulting from dynamic components indicates that the exoskeleton effectively plays a role of load stabilization influencing torso and deltoid muscle activity. This assumption is supported by the more symmetric activity of the ES in the EXO condition. Indeed, asymmetry shown in the NOEXO condition is rather expected from a task requiring walking since gait is commonly asymmetric [50] and muscle activation as well [51]. In the present case, the activity greater for the left ES than right ES in the NOEXO condition may also imply that the load center of gravity is on the side of the body contralateral to the highest activity, which may be associated with the right-hand dominance of the male participants. It is worth noting that, although small, asymmetry in ES activity has been illustrated in standardized lifting tasks; see [52–54]. Another support to load stabilization by the exoskeleton is indicated by the lower activity in the deltoid muscle, which is an upper arm abductor whose action contributes to control the lateral sway of the load in this task [55]. Hence, less lateral load control is required from the torso and shoulder muscles. Overall, the balance tips over a net benefit associated with two concomitant phenomena. One beneficial effect was a lower metabolism in the EXO than NOEXO condition, as indicated by the significantly lower heart rate at the end of the carrying tasks (Fig 6). The other, being the significantly lower perception of discomfort in various body parts associated with a reduction of the physical workload in the EXO condition (Fig 7). Finally, a higher activation in ES for females than males in the NOEXO condition, a gender effect previously observed in other type tasks [51, 53], suggest a more beneficial effect of the exoskeleton for females. Note that a reduction in low back muscle activity has been shown with diverse passive back exoskeletons designs [16, 17, 29, 30]; however, most of them were tested only during dynamic lifting and static forward bend holding.

Although ES EMGs changed significantly during the carrying task, the isometric forward bend holding test didn’t indicate a difference in muscle fatigue post work. Similar tests are commonly used to evaluate muscle fatigue; however, most studies require to produce at least 20% of maximal voluntary contraction for that test. In our study, we standardized the weight to 5kg for all participants. Perhaps this effort was not high enough for most participants to make the test sensitive to muscle fatigue, as a large variability in EMG responses to the test was observed. This latter was most likely associated with the variability in strategy used to maintain the posture. The posture was described as difficult by the participant and despite the standardized control of the back inclination during the test, changes in all joints were possible but not noticeable from the naked eye. Alternatively, since the test was performed immediately after the carrying task the likely long-lasting component of muscle fatigue was not observable due to the interaction of potentiation induced by the dynamic activity of the carrying/walking task and concomitant muscle fatigue [3, 44, 56, 57].

### Upper limbs muscle activity

Deltoid, biceps and brachioradialis muscle activities were significantly reduced after the first two minutes up to the end of the carrying task while using the exoskeleton. Conversely, they generally increase in the NOEXO condition as shown by the respective positive slopes of the linear regressions. In the EXO condition, muscle activation is high during the first 2 minutes. This period may correspond to the classic “adaptation period” (e.g., Bastian, [58]), which in that case is to learn the extent to which the exoskeleton supports and stabilizes the load, leading to a relaxation of the manual interaction with the load thereafter. The negative slopes of the linear regressions in the EXO condition are compatible with a progressive adaptation. Moreover, relative to the median and static activation in each condition the amount of peak muscle activation is clearly higher without than with the exoskeleton. This is particularly true in the deltoid and brachioradialis for both males and females, as illustrated by Figs 3 and 4. It is worth noting that changes in activity associated with adaptation differed between muscles within the first 2 min of the EXO condition. Hence, piecewise regressions with a break point at T1 or T2 could appear arbitrary, although it is not unconceivable that adaptation lag differs between muscles due to their respective role in load control. Hence, despite a conservative approach used to avoid speculations the main effects described above, that would have been enhanced by using a break point, remain significant and support our interpretation. Further detailed analysis is warranted to explain differential adaptation, which is beyond the scope of the present work.

Only a few studies have evaluated upper limb muscle activity during a carrying task with a passive exoskeleton [17, 59]. Their results and ours differ to some extent due to differences in muscle tested and type of exoskeletons used. For example, Theurel et al., [59], used a passive exoskeleton with mechanical arms testing the deltoid and the triceps brachii in relation to the design of their tested exoskeleton. The specificity of the application (e.g., carrying vs lifting) and the exoskeleton designs influence differently the loading/unloading of the muscles [20]. Overall, no clear sex differences were observed for the upper limbs in the tested conditions.

### Heart rate

The much lower increase in heart rate induced by the task when using the exoskeleton indicates an alleviation of the cardiovascular effort in that condition. This result differs from previous studies with passive exoskeletons, as no relevant changes have been reported. This difference can be attributed to their short experimental duration [28] or low level of physical effort [29] since the heart rate reached only 105 bpm in control conditions, which is about 15 bpm less than in our task. Furthermore, these studies have evaluated forward bending or lifting tasks and not carrying. In the present study heart rate without the exoskeleton reached ~120 bpm at the end of the task which corresponds to ~60% of maximum heart rate for our study group, and thus a moderate-hard physical activity [60]. As indicated above the reduction in cardiovascular demand can be associated to the decrease in muscle load demand when using the exoskeleton. Since heart rate is a global measure of metabolism related to energy expenditure [60] the observed reduction while using the exoskeleton may not be attributed solely to a reduction in upper limb muscle activity. It is presumed that other muscles may also benefit from the support provided by this carrying device.

### Discomfort

The concurrent reduction in self-reported discomfort in multiple body segments in the exoskeleton condition corroborates the changes in objective measures and support, like the heart rate results, a global reduction in physical load. Moreover, most participants preferred and will recommend using the exoskeleton for a carrying task. However, less found it comfortable.

### Study limitations

The present study is limited to the carrying task and did not include the lifting and lowering part of common carrying activities. In general exoskeletons are designed to fulfill specific purposes and do not cover all possible tasks performed in real manual material handling jobs. The laboratory simulation was limited to 10 min while real work tasks may require more prolonged and/or repeated activities during the workday. Although not detrimental in the present study, random variability, probably resulting from recording a single lap every two minutes, may be reduced by more frequent recordings. Hence, although long term effects may not be predictable, it can be presumed that any reduction of the physical workload can contribute to a risk reduction of musculoskeletal disorders. Our study included young healthy participants, who represent only a fraction of the general working population. Hence, the impact of the CarrySuit on different age groups needs to be investigated. The Mean-Task method of normalization was selected for the upper limbs; however, different methods of normalization may be used to allow for direct comparison of upper limb muscle activity. Finally, the isometric test used to evaluate low back muscle fatigue was not sensitive enough to detect the effects of the carrying task.

## Conclusions

Upper limb muscle activity decreased over time when using the CarrySuit exoskeleton during 10-min of carrying a 15kg load despite its added weight. Without the exoskeleton, the continuous or significant increase in muscle activities of the upper limbs indicates a constant use of the muscles during the whole task. The exoskeleton also helped reduced ES peak muscle activity with greater benefits for females and contributed to a reduction of asymmetric activation between the left and right ES for males. Taken together these results indicate that the exoskeleton contributes to a lateral stabilization of the load. This benefit is also translated by a reduction of the heart rate increase induced by the task and is corroborated by a lower perceived discomfort when compared to the no exoskeleton condition. Overall, the concurrence of objective and subjective measures supports a global reduction of the physical workload by utilization of the exoskeleton.
